# Treatment and Prevention of Recurrent *Clostridium difficile* Infection with Functionalized Bovine Antibody-Enriched Whey in a Hamster Primary Infection Model

**DOI:** 10.3390/toxins11020098

**Published:** 2019-02-06

**Authors:** Hans-Jürgen Heidebrecht, William J Weiss, Mark Pulse, Anton Lange, Karina Gisch, Heike Kliem, Sacha Mann, Michael W. Pfaffl, Ulrich Kulozik, Christoph von Eichel-Streiber

**Affiliations:** 1 Chair of Food and Bioprocess Engineering, Technical University of Munich, 85354 Freising, Germany; ulrich.kulozik@tum.de; 2 ZIEL Institute for Food & Health, Technical University of Munich, 85354 Freising, Germany; 3 University of North Texas Health Science Center, Fort Worth, TX 76107-2699, USA; William.Weiss@unthsc.edu (W.J.W.); Mark.Pulse@unthsc.edu (M.P.); 4 tgcBIOMICS GmbH, 55411 Bingen, Germany; a.lange@tgcbiomics.de (A.L.); k.gisch@tgcbiomics.de (K.G.); chv.eichel@tgcbiomics.de (C.v.E.-S.); 5 Chair of Animal Physiology and Immunology, Technical University of Munich, 85354 Freising, Germany; kliem@wzw.tum.de (H.K.); michael.pfaffl@wzw.tum.de (M.W.P.); 6 Biosys UK Limited, London SW1H 9BP, UK; sacha.mann@gmail.com; 7 School of Life Science, Technical University of Munich, 85354 Freising, Germany

**Keywords:** *Clostridium difficile* infection, sIgA, bovine milk, prevention of recurrence, hamster model of CDI

## Abstract

Toxin-induced *Clostridium difficile* infection (CDI) is a major disease characterized by severe diarrhea and high morbidity rates. The aim with this study was to develop an alternative drug for the treatment of CDI. Cows were repeatedly immunized to establish specific immunoglobulin G and A titers against toxins A (TcdA) and B (TcdB) and against *C. difficile* cells in mature milk or colostrum. The effect of three different concentrations of anti-*C. difficile* whey protein isolates (anti-CD-WPI) and the standard of care antibiotic vancomycin were investigated in an animal model of CD infected hamsters (6 groups, with 10 hamsters each). WPI obtained from the milk of exactly the same cows pre-immunization and a vehicle group served as negative controls. The survival of hamsters receiving anti-CD-WPI was 50, 80 and 100% compared to 10 and 0% for the control groups, respectively. Vancomycin suppressed the growth of *C. difficile* and thus protected the hamsters at the time of administration, but 90% of these hamsters nevertheless died shortly after discontinuation of treatment. In contrast, the surviving hamsters of the anti-CD-WPI groups survived the entire study period, although they were treated for only 75 h. The specific antibodies not only inactivated the toxins for initial suppression of CDI, but also provoked the inhibition of *C. difficile* growth after discontinuation, thus preventing recurrence. Oral administration of anti-CD-WPI is a functional therapy of CDI in infected hamsters for both primary treatment and prevention of recurrence. Thus, anti-CD-WPI could address the urgent unmet medical need for treating and preventing recurrent CDI in humans.

## 1. Introduction

*Clostridium difficile* (CD)-induced colitis is the leading healthcare-acquired infection, with approximately half a million cases and annual associated costs of $4.8 billion [1]. Even more worrying is the growing number of deaths attributed to *C. difficile.* This number quintupled from 2675 in 2000, to 14,368 in 2007 [2], then doubled again to 29,300 in 2011 [1]—the level at which it has since plateaued in the United States [3]. A major reason for this increase has been the excessive use of antibiotics, accompanied by a growing number of antibiotic-resistant hypervirulent bacterial strains. The disease typically affects elderly patients who have received antimicrobial treatment, especially with broad-spectrum antibiotics that damaged the gastrointestinal microbiota [4]. This has enabled the sporulation of the gram-positive, obligate anaerobic bacterium *C. difficile* as well as its proliferation in the lower gastrointestinal tract [4,5]. Depending on the strain, vegetative cells produce toxin A (TcdA), toxin B (TcdB) and the binary toxins *C. difficile* transferase (CDT) [6]. The most important virulence factors for the pathogenesis of *C. difficile* infection (CDI) are TcdA and TcdB [7,8]. Clinical symptoms range from mild aqueous diarrhea, to life-threatening pseudomembranous colitis and toxic megacolon, to systemic symptoms such as anorexia, nausea, malaise and fever [4,7,9]. To treat these effects, the current standard of treatment is an administration of antibiotics, specifically metronidazole or vancomycin. Although the immediate response to treatment of CDI is typically good, the main issue is the continued destruction of an already damaged gastrointestinal microbiota, leaving the patient susceptible to re-infection. As a result, up to 30% suffer from recurrence within two months after stopping antibiotic treatment [10]. The frequency of multiple recurrences increases to 50–65% after the second recurrence [9].

In summary, there is an urgent need for alternative treatments that avoid the use of antibiotics in order to manage recurrent CDI. One approach is oral treatment, with products obtained from the bovine colostrum of cows with increased concentrations of specific immunoglobulins by immunization. These products offer several advantages over current treatments. The antibodies are polyclonal and can therefore bind to multiple epitopes of the corresponding antigens. Moreover, unlike the more commonly used antibiotics, they are specific to the antigens. Thus, they do not damage the commensal microflora in the gastrointestinal tract and do not contribute to the emergence of new antibiotic-resistant microorganisms [11]. In addition, it is possible to create a pool of antibodies and antibody classes at the same time, which makes it possible to attack both the toxins as well as bacteria simultaneously.

Several animal and human studies summarized elsewhere [12,13] have confirmed the effect of hyperimmune bovine colostrum (HBC) products produced by immunizing cows with different target structures, such as *Escherichia coli* [14,15], influenza virus [16] or rotavirus [17]. Lyerly et al. [18] were the first to show in a hamster model in which HBC with antibodies against TcdA and TcdB was effective in protecting hamsters from CDI. However, all hamsters died within 72 h after the treatment, likely due to the lack of antibodies against *C. difficile*. To solve this problem, van Dissel et al. [11,19] created a whey concentrate with increased concentration of specific immunoglobulins against TcdA, TcdB and *C. difficile*. These studies showed a significant reduction of recurrent CDI in hamster and humans. However, their product was neither characterized nor compared to a standard of care antibiotic treatment and lacks secondary endpoints for understanding of the mechanism of action. In another human study, HBC was as effective as metronidazole [20]. More recently, Sponseller et al. [21] used HBC containing antibodies against recombinant TcdA and TcdB, but not *C. difficile*, to treat CDI in a gnotobiotic piglet model [22]. The group receiving non-immune colostrum developed moderate to severe diarrhea and colitis, while the group treated with HBC had mild to resolved diarrhea and mild or no colitis [21]. Hutton et al. [23] tested another HBC in a mouse model of CDI and showed that the survival rate increased to 77.8% when using a mixture of different antibodies obtained by immunizing cows with multiple single antigens. However, the focus of their study was on TcdB.

A limitation of the use of HBC is that it is available only few hours after birth, which makes collection in large quantities difficult and is why the approach in this study was to use mature immune milk instead of colostrum. The aim was to show that a whey protein isolate (WPI)—obtained from immune milk with predominantly specific secretory immunoglobulin A against the two toxins TcdA and TcdB as well as *C. difficile*—is effective for initial treatment and prevention of recurrent CDI in a hamster primary infection model. In comparison to previous studies in the field, in particular the one by van Dissel et al. [11,19], we aimed at a mechanistic understanding of the treatment result by means of detailed analytical characterization of the WPI material. The immune products were compared for the first time with a standard antibiotic (vancomycin) treatment and the WPI from non-immune milk obtained prior to immunization from exactly the same cows, as opposed to comparing the active product with a similar product of different non-immunized cows as in all previous studies. In addition, as another point of difference to the state of knowledge, we provide ample data of the vegetative and spore counts of *C. difficile* in the intestine and stool to expand the knowledge of the mechanism of action of the bovine antibodies for the treatment of CDI.

## 2. Results

### 2.1. Reduction of TcdA and TcdB Cytotoxicity of WPI Solutions

**Figure 1:**
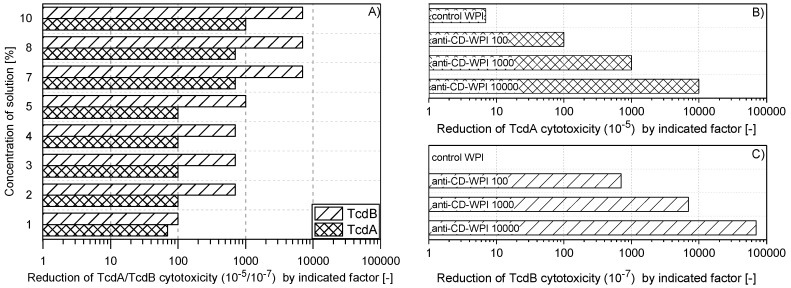
Neutralization capacity of whey protein isolate (WPI) solutions. Defined pure TcdA orTcdB solution could be diluted to a factor of 10^−5^ (10^−7^) on HT29 human colorectal adenocarcinoma cells (or chinese hamster ovary (CHO) cells) to still show cytotoxic activity (cell detachment). This material was used as control. The same toxin solutions were preincubated with anti-CD-WPI for 1 h before determining the remaining cytotoxic activity. The neutralization capacity represented the reduction of the TcdA or TcdB cytotoxicity compared to the effect of the pure toxin exposed exclusively to the buffer. (**A**) shows the neutralization capacity for different concentrations of dissolved WPI powder (weight per volume). Accordingly, percentages of WPI were chosen to define WPI 100 and WPI 1000, and their neutralization capacities were verified, as indicated in (**B**) and (**C**). Control-WPI was prepared from cows before immunization and WPI 10,000 was obtained from the colostrum of immunized cows. Control-WPI, WPI 100, WPI 1000 and WPI 10,000 were used in the hamster protection experiment.

Milk from cows hyper-immunized with the antigens TcdA/TcdB-toxoid and inactivated *C. difficile* strain 630 was collected and processed into single batch of anti-CD-WPI powder. The aim was to test two different concentrations of the same batch of anti-CD-WPI in the hamster model that induce a TcdA neutralization capacity (NC) of 100 and 1000, respectively (see Section 4.4). In order to determine the appropriate WPI dilution, which causes a reduction of the neutralization capacity by a factor of 100 and 1000, the dried powder was dissolved in buffer in different concentrations and tested accordingly (Figure 1A). The 2–5% WPI solutions based on dry weight resulted in a reduction of TcdA activity by a factor of 100 (100 TcdA-NC). Since the TcdB-NC for the 5% WPI solution was still at 1000, the 4% WPI solution was chosen as treatment for WPI 100. To achieve a factor 1000 TcdA-NC, the powder concentration, which correlates with the antibody concentration, had to be increased to 10%. Higher neutralization capacities were only achieved when colostrum was used for WPI preparation. The individual neutralization capacities of WPI 100/1000/10,000 for TcdA and TcdB, as used in the hamster study, are summarized in Figure 1B,C. Along the course of this study, the different treatment groups were classified according to their neutralization capacity for TcdA in order to allow better comparability. The TcdB–TcdA ratio for the neutralization capacities was about 7:1. The negative control WPI, obtained from the same cows as before immunization, had negligible in vitro NC (Figure 1B,C).

### 2.2. Specific Immunoglobulin Titers

**Figure 2:**
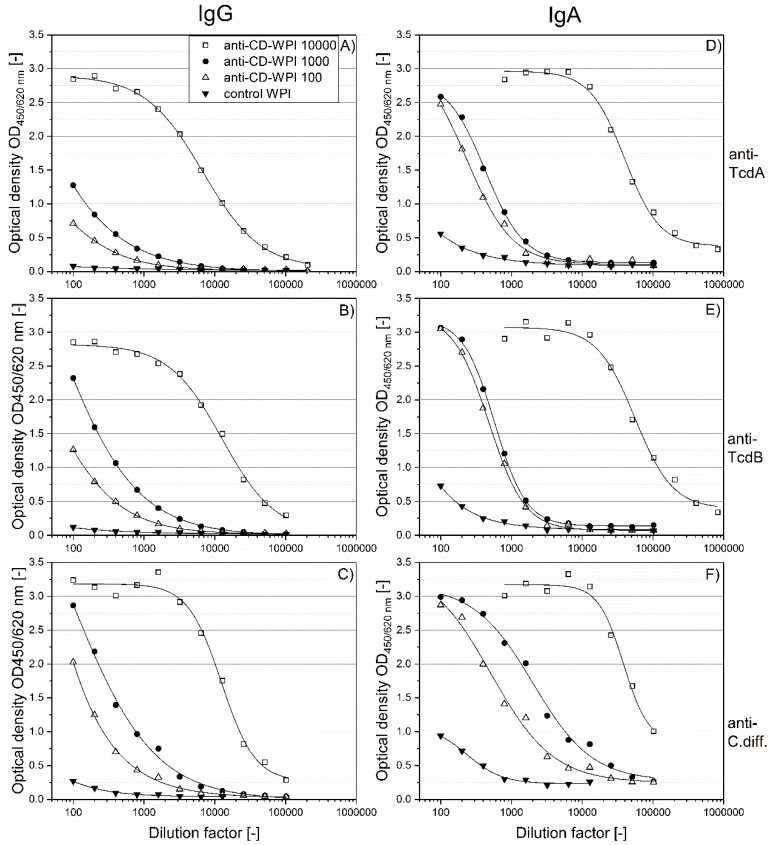
Titration of IgG and IgA antibodies against the respective vaccine antigens. Serial dilutions of WPI 10,000 (open square), WPI 1000 (filled circle), WPI 100 (open triangle) and control-WPI (filled triangle) are plotted against their antibody content. IgG specific for TcdA, TcdB and Cdiff630 (**A**–**C**). IgA specific for TcdA, TcdB and Cdiff630 (**D**–**F**). Curves were adapted using the 4-parameter logistic model.

To determine the specific IgG and IgA contents against the respective antigens of the four defined WPI solutions (Figure 1B,C), serial two-fold dilutions were done using ELISA. As expected and shown in Figure 2, the specific antibody binding capacity for the antigens was in the order anti-CD-WPI 10,000 > anti-CD-WPI 1000 > CD-WPI 100 > control-WPI, for both IgG (Figure 2A–C) and for IgA (Figure 2D–F). Anti-CD-WPI 1000 and control-WPI (10%) had similar total immunoglobulin and total protein concentrations (Table 1) but showed significant differences for all specific immunoglobulin titers. To facilitate the comparison of the specific Ig content of the different WPIs, half-maximal effective concentrations (EC_50_; Table 2) were calculated for the curves in Figure 2.

For WPI 10,000, the ratio of IgA–IgG EC_50_ was 5.9 for anti-TcdA, 4.4 for anti-TcdB and 3.1 for anti-Cdiff630. A similar trend of a higher IgA versus IgG immune response for WPI 1000 and WPI 100 can be seen when comparing values for IgA and IgG summarized in Figure 2.

### 2.3. Prevention of Acute and Recurrent CDI with Anti-CD-WPI

**Figure 3:**
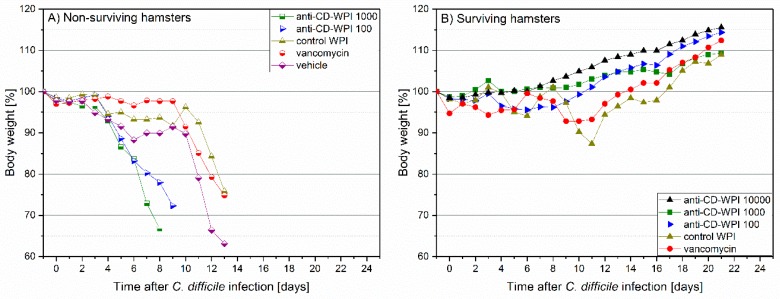
Observation of hamster body weight between days −1 and +20. Mean percentage of body weight relative to day –1. Individual lines summarize the average values obtained for: vehicle (rotated square), vancomycin (circle), control-WPI (triangle up), WPI 100 (triangle right), WPI 1000 (square) and WPI 10,000 (triangle down). Filled symbols represent surviving (S) hamsters (**B**) and half-filled symbols indicate hamsters in individual groups that did not survive (N) due to CDI (**A**).

In the hamster protection study, body temperature and weight were monitored as first signs of CDI development. Surviving animals in all groups had a constant temperature (Figure S1) and showed a continuous average weight gain of 13.0% (mean group range 8.9–15.6%, Figure 3). In hamsters that died of the CDI, we observed a decrease in body temperature of 2.8–6.2% (corresponding to 1.4–3.1 °C, mean of dying animals) and weight loss (mean −17.3%; range −9.3 to −27.0%) (Figure S2). In general, hamsters who died of CDI showed diarrhea and/or had a wet tail shortly before dying. As assessed by necropsy, non-surviving hamsters had discolored and swollen appearance of cecum, ileum and colon with hemorrhagic spots. In contrast, surviving hamsters did not show these symptoms independent of the applied treatment (for details, see Table S1).

**Figure 4:**
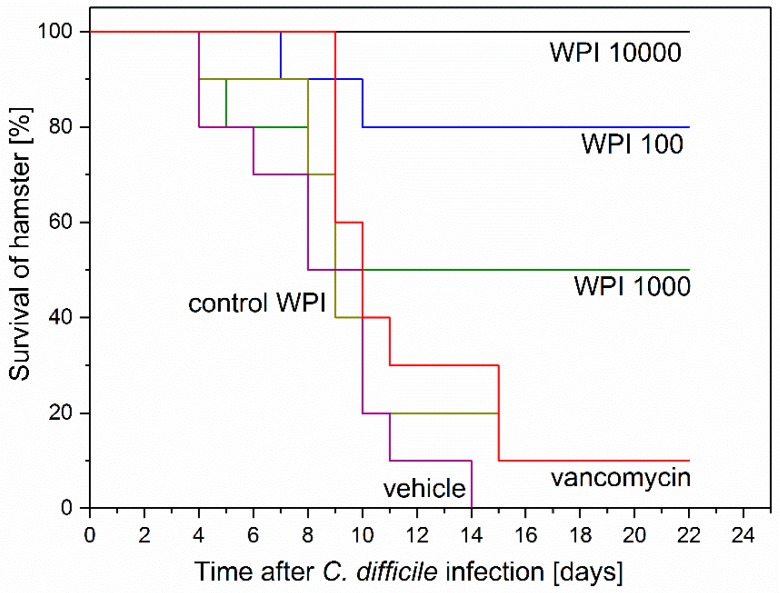
Kaplan–Meier survival curves of hamsters after *Clostridium difficile* infection (CDI) induction and successive treatment. Six groups of 10 hamsters were infected with ~10^2^ *C. difficile* spores 24 h post clindamycin administration. Treatment started at 3 h prior to infection and was performed 3 h after infections and henceforth at 8 h intervals for 75 h before termination. Treatment was as indicated with vehicle (buffer only), vancomycin, control-WPI, WPI 100, WPI 1000 and WPI 10,000. WPI 10,000 survival curve was significantly (log-rank) different from control WPI (*p* < 0.0001), vancomycin (*p* < 0.0001) and vehicle (*p* < 0.00001). WPI 100 survival curve differed significantly (log-rank) from control WPI (*p* < 0.01), vancomycin *p* < 0.01) and vehicle (*p* < 0.001).

The survival of the hamsters, as summarized in Figure 4, was: WPI 10,000 = 100%; WPI 1000 = 50%; WPI 100 = 80%; Control-WPI = 10%; vancomycin = 10%; vehicle = 0%. Thus, the positive control WPI 10,000 treatment protected all hamsters, while untreated vehicle hamsters all died (Kaplan–Meier mean estimator 8.5 ± standard error 1, *p* < 0.00001). The survival of WPI 1000 (14.3 ± 2.5)- and WPI 100-treated hamsters seems somehow inconsistent (50 versus 80% survival) and specifically not consistent with the WPI 10,000 survival (100%). Another observation was that 5 hamsters of the WPI 1000-treated group died early (2 at day 4 and 3 at day 8). Considering 9 out of 10 hamsters in the vancomycin-treated group (11.9 ± 1.3) died of CDI recurrence, treatment with WPI 10,000 reduced recurrence by 90% (*p* < 0.0001), treatment with WPI 100 by 70% (*p* < 0.01) and treatment with WPI 1000 by 40%. Treatment with control WPI resulted in only one hamster surviving (10.4 ± 1.5).

### 2.4. Fecal Vegetative and Spore Colony-Forming Units

**Figure 5:**
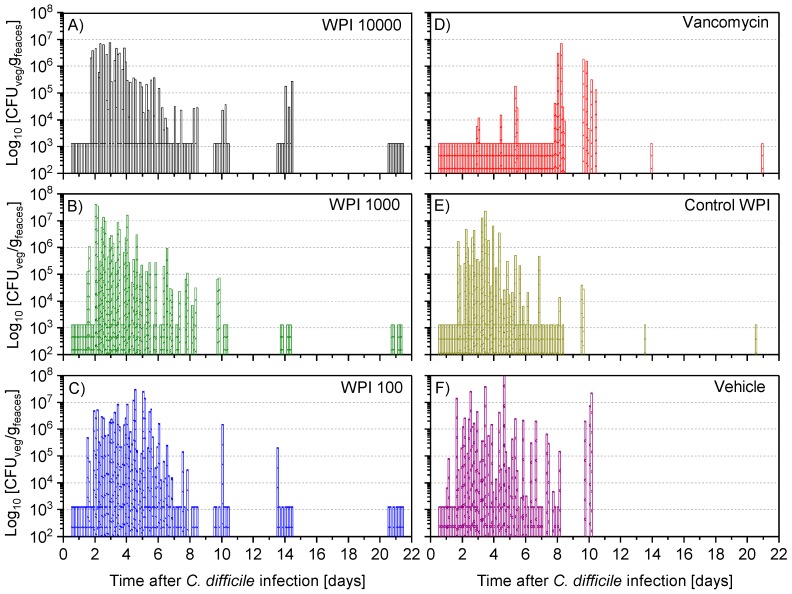
Determination of colony-forming units (CFU) of vegetative *C. difficile* per gram faeces during the following treatments with WPI 10.000 (**A**), WPI 1000 (**B**) WPI 100 (**C**) vancomycin (**D**), control-WPI (**E**), vehicle (**F**). For each hamster fecal samples were taken twice daily on days 1–6 and on days 7, 8, 10, 14 and 21 to determine the CFU of vegetative cells (CFU-veg) of *C. difficile* in indicated treatments. Up to 20 bars of one group are displayed for the individual animals (on day 1–6 the 2 samples are superimposed). Accordingly, on day 20 there are 10 bars in the WPI 10,000 group, 1 in the vancomycin/control-WPI group and 0 after vehicle treatment. CFU/g detection limit was 3.11 log_10_.

**Figure 6:**
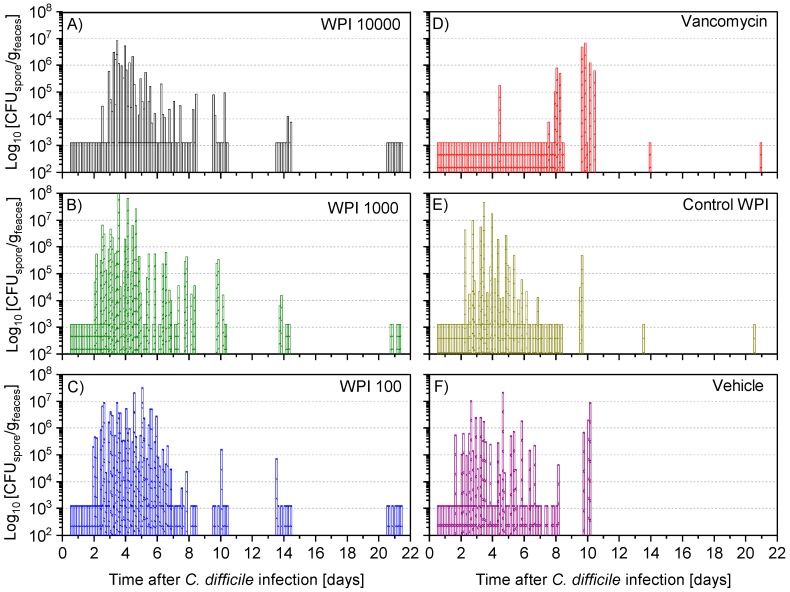
Determination of colony-forming units of spores (CFU-spore) of *C. difficile* per gram faeces during the following treatments with WPI 10.000 (**A**), WPI 1000 (**B**) WPI 100 (**C**) vancomycin (**D**), control-WPI (**E**), vehicle (**F**). For each hamster fecal samples were taken twice daily on days 1–6 and on days 7, 8, 10, 14 and 21 to determine CFU counts of the spores of *C. difficile*. CFU/g detection limit was 3.11 log_10_.

After 12 to 24 h post-infection, the vegetative (Figure 5) and spore colony-forming unit (CFU) counts (Figure 6) were still below the detection limit in almost all hamsters. At 36 h after infection, the vegetative CFU count was greatly increased (Figure 5) in all groups except the vancomycin-treated group. At the same time, the number of spores—with the exception of 5 (of 60) hamsters—was still below the detection limit (Figure S3). Vegetative and spore counts were significantly lower in the vancomycin group until the fourth day, while there was no difference between the five remaining treatment groups. Until day 7 the difference in CFU in the vancomycin-treated group was still significantly lower except for the WPI 10,000-treated group, which had similarly low numbers. However, on day 10, both the vegetative and spore counts were significantly higher in the vancomycin-treated group compared to the 3 groups treated with WPI obtained from immunized cows (pairwise statistical comparison is given in Appendix A). For WPI 10,000, WPI 1000, WPI 100 and control-WPI, the general course was a peak of fecal vegetative and spore *C. difficile* CFU counts at 2–6 days, and independent of the treatment, a subsequent drop below the detection limit in surviving hamsters by the end of the 21-day study. Moreover, the number of spores in the intestine—the site of action of CDI—was below the detection limit in all surviving hamsters except one in the control-WPI group (Figure S4). In contrast to the latter, the mean vegetative and spore counts in the last fecal sample before the hamsters died due to CDI was 5.9 × 10^6^ g/feaces (whisker range 2.2–6.3 × 10^6^) and 5.4 × 10^6^ g/feaces (range 2.3–8.1 × 10^6^), respectively (Figure S5). On the day of death, the analogous concentrations in the cecal fluid were 1.82 × 10^7^ (whisker range 3.5 × 10^6^–5 × 10^7^) and 4.7 × 10^6^ (whisker range 6 × 10^4^–10^7^), respectively. Such counts were detected independently of the previous treatment. Groupwise, individual numbers of CFU in the cecal fluid are given in Figure S6.

## 3. Discussion

The objective of vaccinating cows with *C. difficile* antigens is to produce hyperimmune milk containing a pool of various polyclonal antibodies and antibody classes against these antigens, which in turn can be used to treat and prevent toxin-induced CDI in humans. Both toxins TcdA and TcdB are known to induce CDI in humans [4]. Hence, the WPI were characterized by their NC against the two toxins, as well as their ability to bind these antigens and *C. difficile*.

The comparison of the anti-CD-WPI 1000 with the control-WPI clearly showed that it is possible to specifically increase the antibody titer for *C. difficile* and the toxins TcdA and TcdB in mature milk by immunizing cows, retain this function during processing into WPI and protect against CDI in vivo. Both products had similar total immunoglobulin and total protein concentrations but exhibited significant differences for all specific IgG and IgA immunoglobulin titers. This is in agreement with Young et al. [11], who showed that the total immunoglobulin concentration remained constant before and after immunization, while the specific concentration against the respective antigens increased for IgA. In addition to their study, this work also demonstrated an increase in specific IgG antibodies in milk. Another difference to previous studies is that we used the milk of the same cows before and after the immunization, as opposed to comparing the active product to a similar product from another herd of non-immunized cows. The effect of immunization to induce a specific in vivo activity was thus more clearly demonstrated. Probably such a comparison was not possible in previous studies for practical reasons, since the time interval to collect colostrum from the same cow is at least about one year between calf births.

The results show that the applied immunization protocol predominantly enhanced the specific IgA response in colostrum and mature milk, which is in agreement with Young et al. [11], but in contrast to all other studies that focused only on the main immunoglobulin class IgG. Although the concentration of IgG in bovine milk is about 8–10 times higher than IgA, it is reversed in human breast milk, where IgA accounts for more than 80% of the total immunoglobulin fraction. Secretory IgA is the dominant immunoglobulin class in mucosal secretions and therefore of great importance for the defense mechanism of the first line. Its main function is to prevent mucosal infections by agglutination of microbes and neutralization of toxins [24]. Thus, the high specific IgA titers are particularly important for the treatment of CDI, because the site of action is the intestinal epithelial layer [5]. In addition, the secretory component bound to the dimer IgA protects the antibody from proteolytic degradation [25,26], while IgG degradation during the passage through the gastrointestinal tract is more than 95% [27].

Clindamycin-induced CDI in hamsters is the most commonly used in vivo model for assessing the efficacy of potential agents, including antibiotics and toxin antibodies based on similar pathophysiological characteristics observed in humans [28]. The *C. difficile* concentration in the stool of the hamsters who died of CDI (6.77 log_10_ CFU/g) was similar to the fecal load of CDI-positive patients (6.67 log_10_ CFU/g, interquartile range 5.57–7.54 log_10_ CFU/g) [29]. We used a primary infection model with a treatment period of 75 h and monitored the survival of 6 different treatment groups of 10 hamsters each for 21 days. Our HBC (WPI 10,000) resulted in a survival rate of 100%. The only other group that ever observed complete survival in the treatment of CDI was Lyerly et al. [18]. However, all hamsters died within 72 h after discontinuation of their HBC treatment, most likely due to the lack of antibodies against *C. difficile*. In contrast, none of the hamsters died after discontinuing treatment with our HBC containing antibodies against the toxins and *C. difficile*. This also differs from the results of Hutton et al. [23], where the animals had access to the active substances in the drinking water during the entire prophylaxis study. This led to a survival of 77.8% when mice were treated with the best HBC mixture [23].

**Figure 7:**
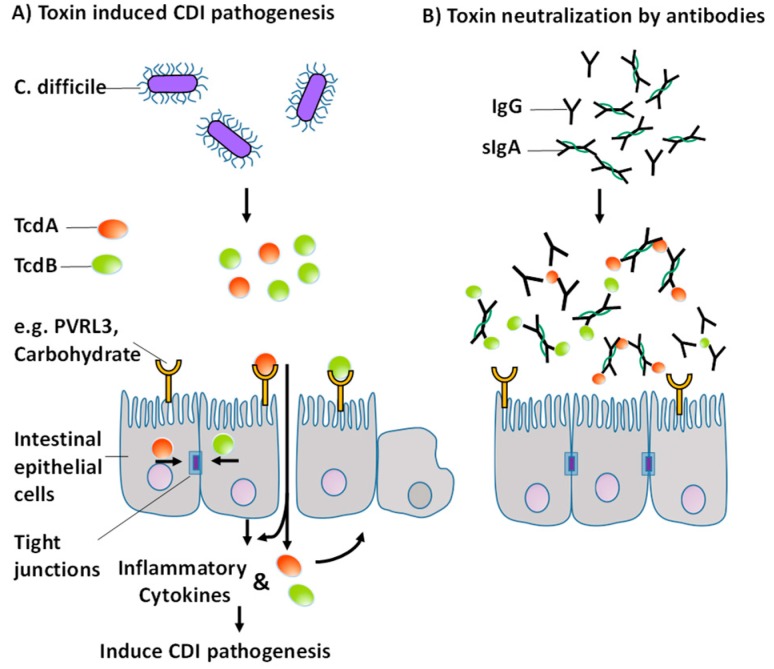
Toxin-induced pathogenesis of CDI modified from [34] (**A**) Proposed mechanism for acute treatment of CDI by specific polyclonal antibodies against TcdA and TcdB (**B**).

Antibodies can inactivate foreign bodies through various mechanisms of action. These include, among others, opsonization, activation of the complement system, agglutination, preventing adhesion and direct neutralization. TcdA is a protein with 308 kDa and TcdB is a protein with 269 kDa. Therefore, the toxins have about the same size as immunoglobulins IgG (160 kDa) and sIgA (400 kDa), at least in comparison to *C. difficile*, which has a size in the range of 1 micron. The proposed mechanism responsible for the acute treatment and initial survival is direct neutralization of the toxins. Pathogenesis is suppressed by binding of the antibodies to the active epitopes of the toxins and sterically preventing binding of the toxins to receptors or carbohydrates at the epithelial barrier (Figure 7). Remarkably, hamsters treated with our immune products survived not only the initial phase, but also throughout the study, although they were treated for only 75 h. It is likely that the survival of WPI 1000 and WPI 100 would have been higher during prolonged administration.

A core objective of this work was to compare our immune products with a standard antibiotic therapy, since such a comparison had not been done in previous studies. Our results show that vancomycin suppresses growth of *C. difficile* from the onset of treatment and initially protected the hamsters. However with a time lag of six days (from the end of the antibiotic treatment) the hamsters began to die at day nine, consistent with other studies [30,31,32]. According to our CFU vegetative/spore data, vancomycin must be metabolized under the active dose until then so that *C. difficile* spores were able to germinate and produce toxins, which led to the death of the hamsters. In contrast, the initial *C. difficile* growth was not suppressed for the other treatment groups as was observed during treatment with another HBC in mice [23]. In contrast to previous reports, however, we observed a decline in cell growth after about 60 h in hamsters that were treated with our hyperimmune WPI-products. Thus the present work shows for the first time that hyperimmune WPI treatment interferes with bacterial growth and that an initial *C. difficile* outgrowth is followed by suppression of growth on the long run We expect that this effect will not be strain specific since the WPI contains polyclonal antibodies that will bind to multiple *C. difficile* epitopes. The reasons for the decline in growth should be studied in more detail. Some possible explanations, however, are the antibody binding to cells and spores, the support of the regeneration of the natural intestinal microflora and activation of the immune system of the host. It is well-known that a disturbed microflora leaves patients susceptible to the recurrence of CDI [4,33]. The antibodies are only specific for their antigens, thus they do not further alter the already damaged microflora. Therefore, the recovery of the native microflora can begin immediately after the initial destruction with clindamycin. A native microbiota prevents *C. difficile* growth [5]. Moreover, the inhibition of the *C. difficile* toxin activity by the anti-toxin antibodies could protect the colonic mucosa from further damage, thereby allowing regeneration of the epithelium, which in turn leads to environmental changes not favoring *C. difficile* growth. This should be confirmed by histopathological research in future investigations. In addition, the natural function of IgG is to initiate the classical complementary pathway [24]. The host´s immune system may also contribute to *C. difficile* growth reduction. These factors might also contribute to the unexpectedly lower protection of the anti-CD-WPI 1000 compared to WPI 100. From this comparison of the vancomycin- and WPI-treated groups, a new treatment proposal for oral applications in humans can be derived, namely that the products should be given in parallel. After diagnose of CDI, treat the acute phase with a pulse of vancomycin, for example, for 2–3 days (instead of 10 days as it is currently applied in the hospital) and WPI simultaneously. Vancomycin will suppress the CDI acute phase and antibodies against the toxins and bind the toxins that are initially produced after the administration of antibiotics (when changing from the vegetative state to spore). After the end of vancomycin application (day 2–3) continue with WPI treatment only. Such treatment may protect against late CDI effects and inactivate spores (which is not the case for vancomycin only). This means that the dose of antibiotics can be significantly reduced, which reduces the damage to the microbiome and accelerates the regeneration of microbial diversity, thus reducing recurrence rates. We are particularly confident that the combined treatment is effective for the second and higher episode of CDI. In addition, our product, with an excellent safety profile, could be used to prevent CDI in susceptible patients based on known risk factors such as surgery and clindamycin application or inflammatory bowel disease. The product might therefore address an unmet medical need.

## 4. Materials and Methods

### 4.1. Preparation of Whey Protein Isolates

TcdA and TcdB were produced by cultivation of *C. difficile* strain VPI10463, and cell material was produced from *C. difficile* 630; both substances were inactivated before application to the cows according to van Dissel et al. [19]. A heard of six non-pregnant Holstein Friesian cows and a second herd of four pregnant Holstein Friesian cows were repeatedly immunized with inactivated whole *C. difficile* cells and TcdA and TcdB toxoids as described by van Dissel et al. [19]. Immunization of pregnant and non-pregnant cows was carried out under approval of the government of Upper Bavaria (AZ 55.2-1-54-2532.6-17-12). Standard milking techniques were used to collect the immune milk. Fat was removed by centrifugation to <0.1% and microorganisms and casein micelles were removed by microfiltration to obtain a whey containing the immunoglobulins in their native state. Whey proteins were concentrated by ultrafiltration, and lactose and salt concentrations were adjusted by addition of water to obtain a whey protein isolate (anti-CD-WPI) with a protein content of ≥90% [35]. Control whey was obtained from milk of the same cows prior to immunization and frozen at −18 °C until it was equally processed to the control-WPI. Colostrum from the first two milkings was collected from the second cohort of immunized pregnant cows and served as a positive control. Processing of colostrum and immune milk was generally identical, with the exception that the colostrum was diluted with water to 10% dry matter to facilitate casein removal by microfiltration. The liquid WPI solutions obtained were freeze-dried as described previously [36]. In total, 44 doses (1 dose for 10 hamsters, 11 time points, 4 treatment groups) were prepared by weighing equivalent amounts of powder into sterile tubes. The final concentration was 10% except for WPI 100 (4%) after adding 11 mL buffer immediately before application. The buffer contained 1 M NaHCO_3_ /Enfamil^®^ Infant (Mead Johnson Nutrition, Chicago, IL, USA). Both substances are reported to improve the gastrointestinal passage of the proteins [18,19,37].

### 4.2. Whey Protein Characterization

The total IgG, IgA and IgM were measured with ELISA-kits cat. No. E10-118, E10-121 and E10-101 (Bethyl Laboratories, Montgomery, TX, USA) following the manufacturer’s instructions. Total protein content and dry matter was measured according to Marx and Kulozik [38].

### 4.3. Determination of IgG and IgA against TcdA, TcdB and C. difficile 630

Six separate ELISA-tests were applied to determine the specific IgG or IgA levels against the major vaccine antigens toxin A, toxin B and *C. difficile* 630. ELISA-kits (TGC-E101-1, TGC-E102-1, TGC-E103-1) were purchased from tgcBIOMICS GmbH (Bingen, Germany) and carried out according to the manufacturer’s description. Pure buffer served as negative control and, as an internal positive control, whey from immune colostrum was used.

### 4.4. Inhibition of Cytotoxicity of TcdA and TcdB

Two separate cytotoxicity tests were performed to determine the neutralizing activity of the WPI solutions. Toxins were obtained from *C. difficile* strain VPI10463. The cytotoxic activity induced by cytotoxin TcdB was measured on Chinese hamster ovary cells (CHO-K1, ECACC 85051005). Because the enterotoxin TcdA applied to CHO cells shows an activity approximately 1000 times lower than toxin B, adherent human colorectal adenocarcinoma cells (HT29, ECACC 91072201) were used for the measurement of TcdA neutralization capacity, which increased the sensitivity of the measurement. The assay was performed as previously described by Moos and von Eichel-Streiber [39] with the following modifications.

In the first row of a 96-well plate, the biological activity of the pure toxin solutions of TcdA or TcdB on the respective cell detachment was measured by tenfold series dilutions. The TcdA solution induced cell rounding to a dilution of 10^−5^ dilution on HT29 cells, the TcdB solution induced cell rounding to a dilution of 10^−7^ on CHO cells. In the remaining rows of the 96-well plate, the WPI solutions, which had been incubated for 1 h at 37 °C with the same toxin solution, were added to the respective adhering cells. A preliminary result was obtained after 3 h and the final value after exposure of the mixtures to the cells overnight. The final readout was expressed as a factor of the difference between the pure toxin activity and the remaining activity after antitoxin exposure. The factor is called neutralization capacity (NC). An NC of 1000, for example, indicates that the antitoxin (IgG and IgA antibodies against the respective toxin) contained in a corresponding WPI solution reduces the cytotoxicity of the respective toxin in the cytotoxicity assay by three logs. In this case, a TcdB-NC of 1000 means that pure TcdB exerts cytotoxic activity up to a dilution of 10^−7^, whereas the TcdB-WPI preincubated solution is cytotoxic only up to a TcdB dilution of 10^−4^.

### 4.5. Hamster Treatment

Sixty male golden Syrian hamsters, (89.5–114 g, mean 100.4 g) purchased from Envigo (Harlan) laboratories (Houston, TX, US), were housed in individual cages with free access to food and water. All procedures were conducted in accordance with the approved protocol of the Institutional Animal Care and Use Committee (IACUC-2016-0015). The animals were acclimatized for five days prior to the study in the biosafety level laboratories at University of North Texas Health Science Center. Twenty-four hours after intraperitoneal clindamycin injection (10 mg/kg body weight), 6 groups of 10 hamsters each were challenged with ~10^2^ spores of *C. difficile* strain 630 by gastric tube. The different groups were: anti-CD-WPI 10,000, anti-CD-WPI 1000, anti-CD-WPI 100, control-WPI, vancomycin and vehicle. The groups were distinguished according to their NC against TcdA (as described above). Animals were given 1 mL WPI-solution/100 g body weight by oral gavage for 75 h at the same times as described (−3, 3, 11, 19, 27, 35, 43, 51, 59, 67 and 75 h) [19]. This design was chosen to observe whether the antibodies could prevent colonization of *C. difficile* in the intestine. Vancomycin was applied after 3, 27, 51 and 75 h with 2 mg/100g body weight. The vehicle group received the pure buffer solution. Hamsters were monitored daily for disease symptoms and loss of body temperature and weight. In addition, the texture of the stool was inspected and fecal pellets were collected for further analysis. Animals either succumbed to the disease or were euthanized for gross necropsy at the end of the study. Post mortem, the colon was visually inspected for swelling (mega-colon) and other macroscopic pathological lesions. The small intestine and caecum were examined for signs of swelling, inflammation or hemorrhage.

### 4.6. Fecal Total CFU Detection and Spore Detection

The number of colony-forming units (CFU) of *C. difficile* was determined using either 1 ml cecal fluid on the day of death (day 21, for surviving hamsters) or 5–8 fecal pellets for each hamster twice daily on days 1–6 and on days 7, 8, 10, 14 and 21. The weight was determined, 2 mL of sterile phosphate buffered saline (1X PBS) was added and the mixture was homogenized for 15 s. An additional 3 mL sterile 1X PBS was added and the solution was allowed to settle for 30 s to remove larger solid particles. Aliquots of 200 µl of the supernatant were transferred to row A of a 96-well plate and thereafter serially diluted tenfold into rows B to H. Eight µL of each serial dilution were plated on *C. difficile* selective agar cycloserine cefoxitin fructose supplemented with w/w 0.1% sodium taurocholate. Inoculated plates were incubated under anaerobic conditions for 48 h at 37 °C. Plates that contained 5 to 50 colonies per inoculum were used to calculate the CFU number. For spore detection, vegetative cells were inactivated by heating the mixture to 65 °C for 30 minutes after the homogenization step and then treated accordingly. The detection limit was 3.11 log_10_ CFU/g for fecal and 2.7 log_10_ CFU/mL for cecal samples.

### 4.7. Data Regression and Statistical Analysis

Kaplan–Meier survival curves were evaluated with log-rank test. Data were considered significant at *p* value of <0.05 [40]. The dose-response curves were adjusted using the 4-parameter logistic model according to the following formula: y = A2 + (A1 − A2)/(1 + (x/x_0_)^p), with the parameters A1 the minimum initial value, A2 the maximum final value, x_0_ the center and the power p = 3 of the sigmoidal curve. To determine the corresponding values the iteration algorithm according to Levenberg–Marquardt [41] was used. The median effective concentration (EC_50_ = x_0_) was defined as the concentration at which the immunoglobulins exerts half of their maximal optical density(OD). OriginPro v2017 (OriginLab Corporation, Northampton, MA, USA) software was used for these statistic evaluations. Since microbial growth rarely follows normal data distribution, a nonparametric pairwise test is required to determine significant differences between the *C. difficile* CFU counts of the treatment groups. An R script [42] based on the non-parametric Mann–Whitney Test [43] was used. The pairwise significance values obtained were corrected for multiple testing with the Benjamini–Hochberg method [44].

## Figures and Tables

**Table 1:** Composition and half-maximal effective concentration (EC_50_) for different whey protein isolates (WPI).

Parameter	WPI 10,000	WPI 1000	WPI 100	Control-WPI
Total IgG [mg/mL]	57.7	3.5	1.55	3.81
Total IgA [mg/mL]	3.01	0.43	0.17	0.43
Total IgM [mg/mL]	5.81	0.89	0.39	0.81
Total protein [%]	96.78	91.24	91.24	90.1
Dry matter [%]	98.09	94.15	94.15	92.51

**Table 2:** Half-maximal effective concentration (EC_50_) for different whey protein isolates (WPI).

EC_50_	WPI 10,000	WPI 1000	WPI 100	Control-WPI
IgG TcdA	6793 ± 291	NA	NA	NA
IgG TcdB	12665 ± 1312	NA	NA	NA
IgG *C. difficile*	12445 ± 124	NA	NA	NA
IgA TcdA	39882 ± 2737	428 ± 17	230 ± 47	NA
IgA TcdB	55708 ± 6601	578 ± 11	480 ± 18	NA
IgA *C. difficile*	38222 ± 7014#	2021 ± 292	516 ± 146	235 ± 51

± represents standard deviation. Values and standard deviation rounded up to the last digit. NA = not available because of the non-sigmoidal progress EC_50_ calculation was not possible. Pearson R^2^ was >0.99 for all fits, except #0.97.

**Figure A1:**
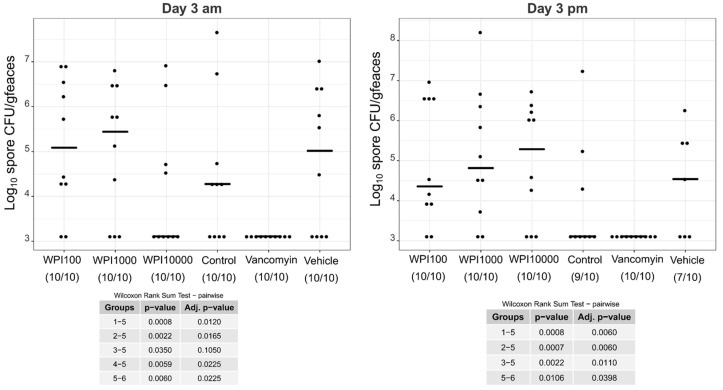
Log_10_-CFU spore counts of various treatment groups on day 3. WPI (whey protein isolate) is characterized by its neutralization capacities against toxin A (100, 1000, 10,000). Control-WPI was produced from the cows before immunization and vehicle was only the buffer. CFU/g detection limit was 3.11 log_10_.

**Figure A2:**
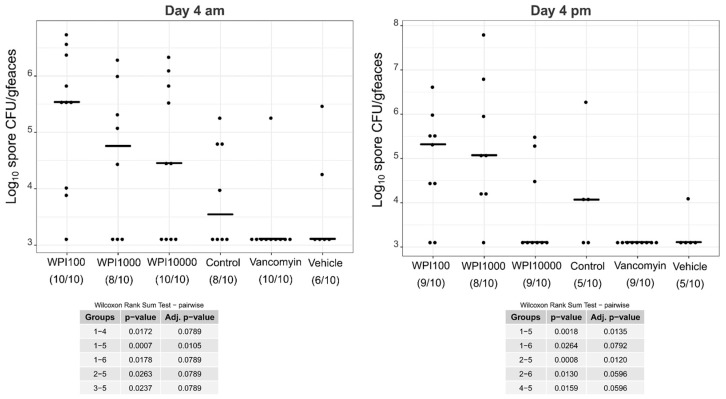
Log_10_-CFU spore counts of various treatment groups on day 4. WPI (whey protein isolate) is characterized by its neutralization capacities against toxin A (100, 1000, 10,000). Control-WPI was produced from the cows before immunization and vehicle was only the buffer. CFU/g detection limit was 3.11 log_10_.

**Figure A3:**
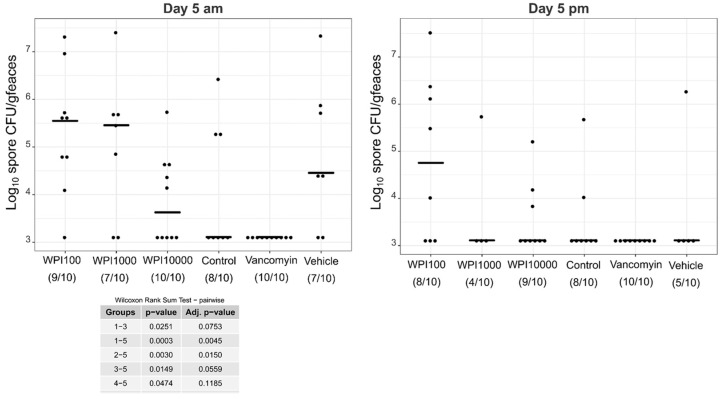
Log_10_-CFU spore counts of various treatment groups on day 5. WPI (whey protein isolate) is characterized by its neutralization capacities against toxin A (100, 1000, 10,000). Control-WPI was produced from the cows before immunization and vehicle was only the buffer. CFU/g detection limit was 3.11 log_10_.

**Figure A4:**
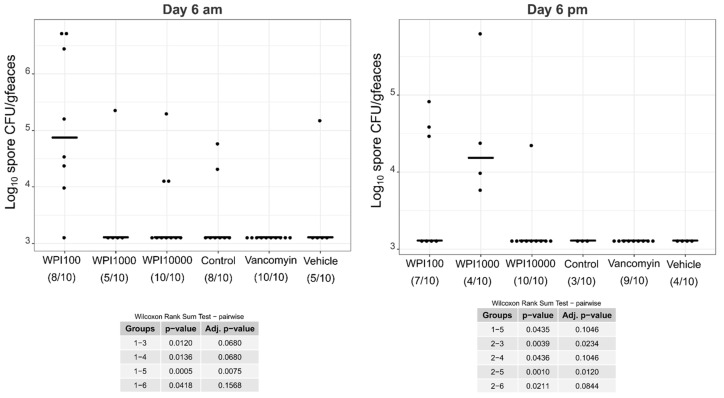
Log_10_-CFU spore counts of various treatment groups on day 6. WPI (whey protein isolate) is characterized by its neutralization capacities against toxin A (100, 1000, 10,000). Control-WPI was produced from the cows before immunization and vehicle was only the buffer. CFU/g detection limit was 3.11 log_10_.

**Figure A5:**
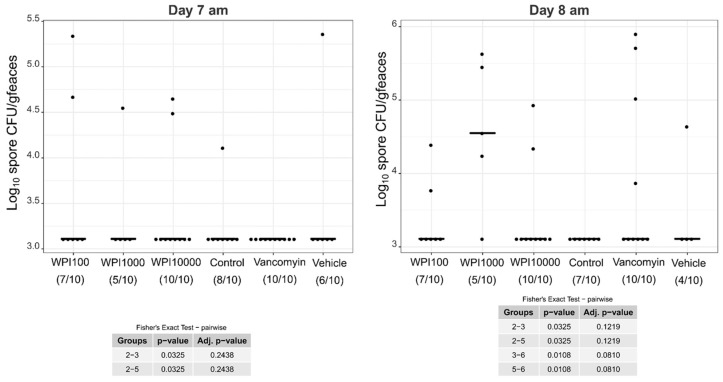
Log_10_-CFU spore counts of various treatment groups on days 7–8. WPI (whey protein isolate) is characterized by its neutralization capacities against toxin A (100, 1000, 10,000). Control-WPI was produced from the cows before immunization and vehicle was only the buffer. CFU/g detection limit was 3.11 log_10_.

**Figure A6:**
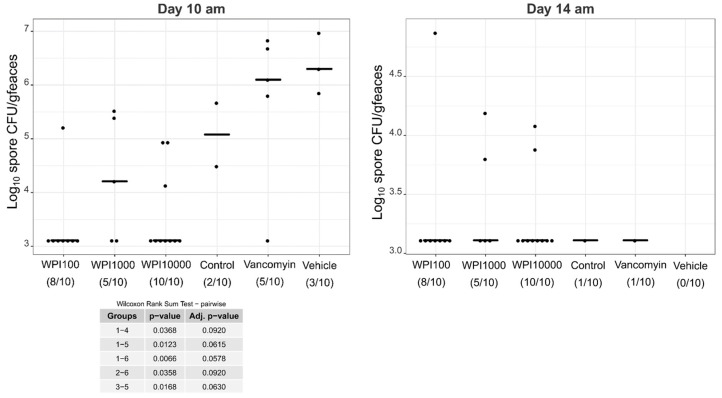
Log_10_-CFU spore counts of various treatment groups on days 7–8. WPI (whey protein isolate) is characterized by its neutralization capacities against toxin A (100, 1000, 10000). Control-WPI was produced from the cows before immunization and vehicle was only the buffer. CFU/g detection limit was 3.11 log_10_.

**Figure A7:**
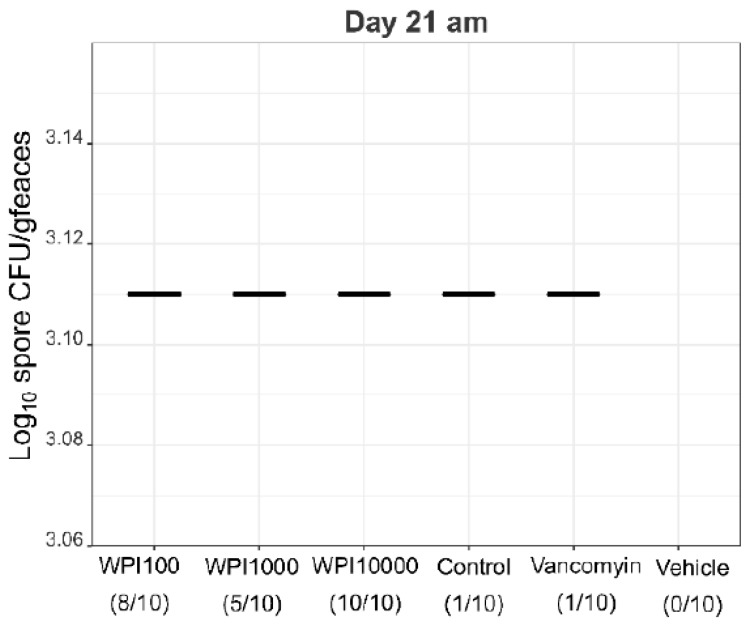
Log_10_-CFU spore counts of various treatment groups on days 7-8. WPI (whey protein isolate) is characterized by its neutralization capacities against toxin A (100, 1000, 10,000). Control-WPI was produced from the cows before immunization and vehicle was only the buffer. CFU/g detection limit was 3.11 log_10_.

## Supplementary Materials

The following are available online at http://www.mdpi.com/2072-6651/11/2/98/s1, Figure S1 Average body temperature of individual groups, Figure S2 Average body weight ± standard deviation related to the average weight on day 1 before any treatment. Figure S3 Vegetative and spore) colony-forming units (CFU) of *C. difficile* per gram faeces of during the treatment with whey protein isolates 36 h post infection. Figure S4 Spore counts CFU/ml in the cecal fluid at the day 21. Figure S5 CFU/g feces or mL fecal fluid at the day of death of hamster who succumbed to the disease regardless of applied treatment. Figure S6 CFU/ml cecal fluid at the day of death of hamster who succumbed to the disease for the different applied treatments. Table S1 Necropsy observation of cecum, ileum and colon.

## Appendix A

The appendix shows a pairwise statistical comparison of the CFU spore counts of *C. difficile*.

Since microbial growth rates rarely follows the normality of data distribution, a nonparametric pairwise test is required to determine significant differences between *C. difficile* CFU spore counts of the treatment groups. An R script [42] based on the non-parametric Wilcox Rank Sum Test [43] was used. Significance values are only displayed for data whose *p*-value is <0.05. This means that if no *p*-values are given in the tables below the graphs between the groups, there was no significant difference. The obtained pairwise significance *p*-values were corrected for multiple testing with the Benjamini-Hochberg method [44]. The number of CFU spores for the day 1 am until day 2 pm are not displayed because there were no significant differences. The number in parentheses indicates the number of samples at that time, and generally correlates with the number of hamsters surviving at that time. A few samples are missing because no sampling was possible.
