# Supplementary material for: Treatment and Prevention of Recurrent Clostridium difficile Infection with Functionalized Bovine Antibody-Enriched Whey in a Hamster Primary Infection Model
Source: Toxins (Basel). 2019 Feb 6;11(2):98. doi: 10.3390/toxins11020098 (PMC6409564; doi:10.3390/toxins11020098)
Supplement: Supplementary file 1 [file toxins-11-00098-s001.pdf]

Supplementary data regarding the treatment and prevention of recurrent *Clostridium difficile* infection with functionalized bovine antibody-enriched whey in a hamster primary infection model

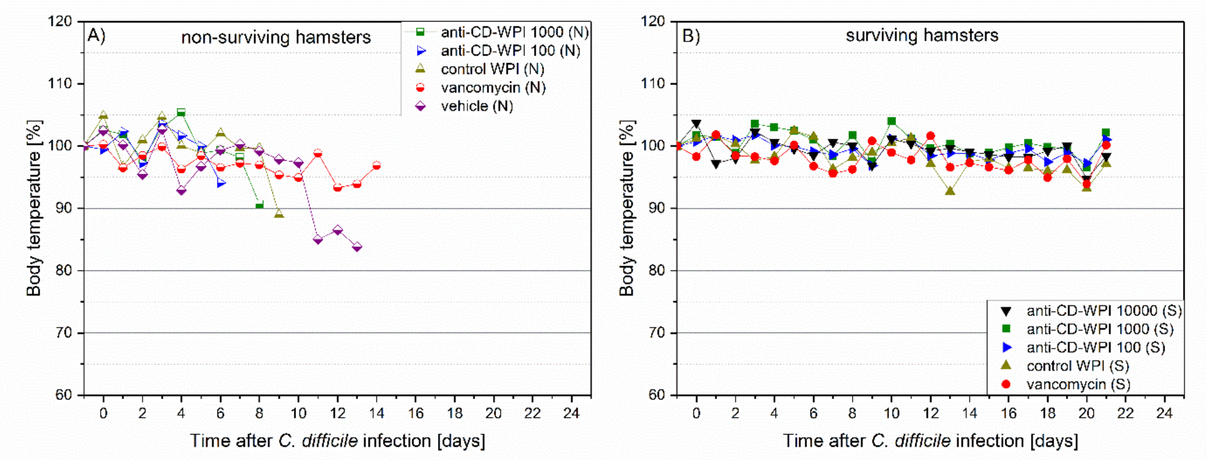

Figure S1 Observation of hamster body-temperature between days −1 and +20: Mean percentage of body temperature relative to day −1. Individual lines summarize the average values obtained for: vehicle (rotated square), vancomycin (circle), control-WPI (triangle up), WPI 100 (triangle right), WPI 1,000 (square), and WPI 10,000 (triangle down). Filled symbols represent surviving (S) hamsters (B) and half-filled symbols indicate values of hamsters in individual groups that did not survive (N) due to CDI (A).

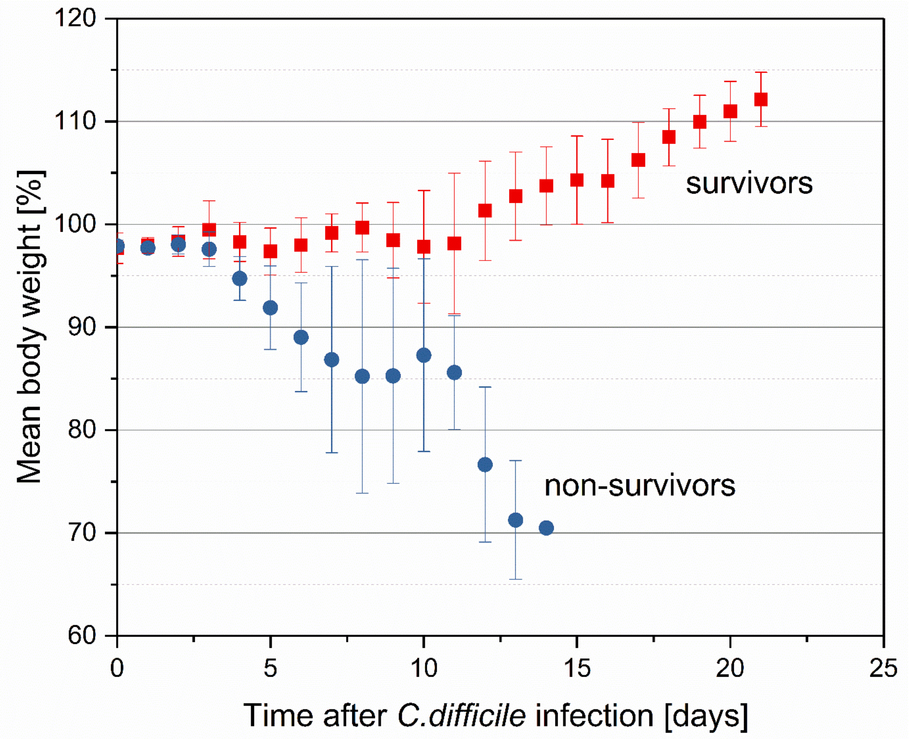

Figure S2 Mean body weight ± standard deviation related to the average weight on day −1 before any treatment. Squares (■) represents surviving hamster of all groups and circles (●) the hamster succumbed to the disease of independent of the applied treatment.

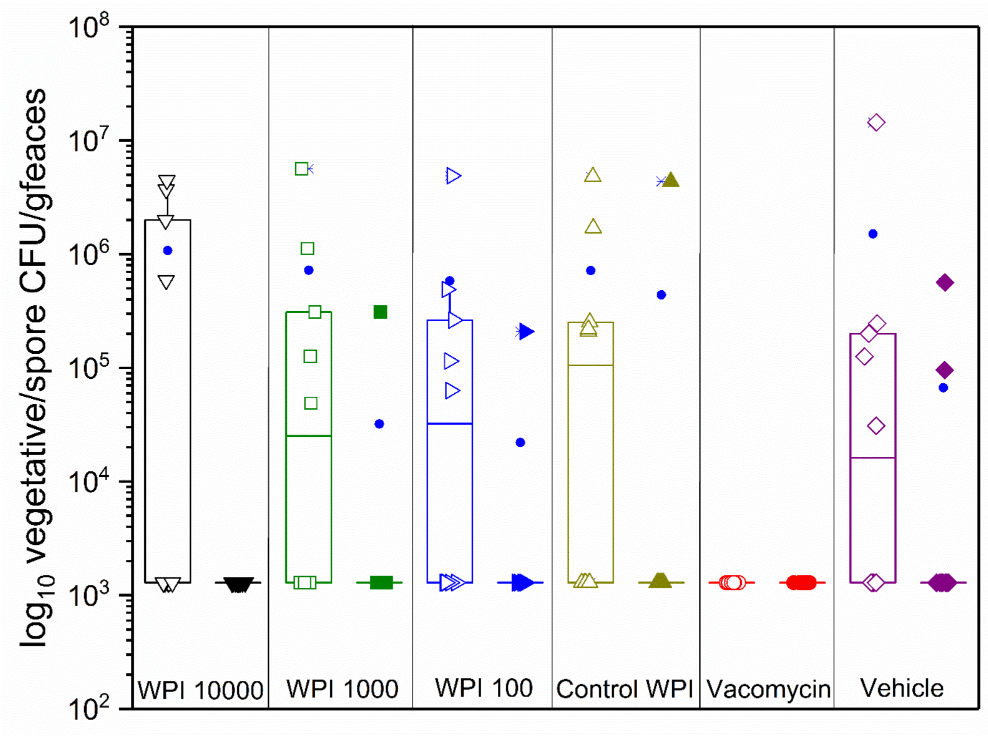

Figure S3 Vegetative (open symbols) and spore (filled symbols) colony-forming units (CFU) of *C. difficile* per gram faeces of during the treatment with whey protein isolates 36hours post infection. The blue dot represents the mean. Detection limit was 3.11 log_{10} CFU/g.

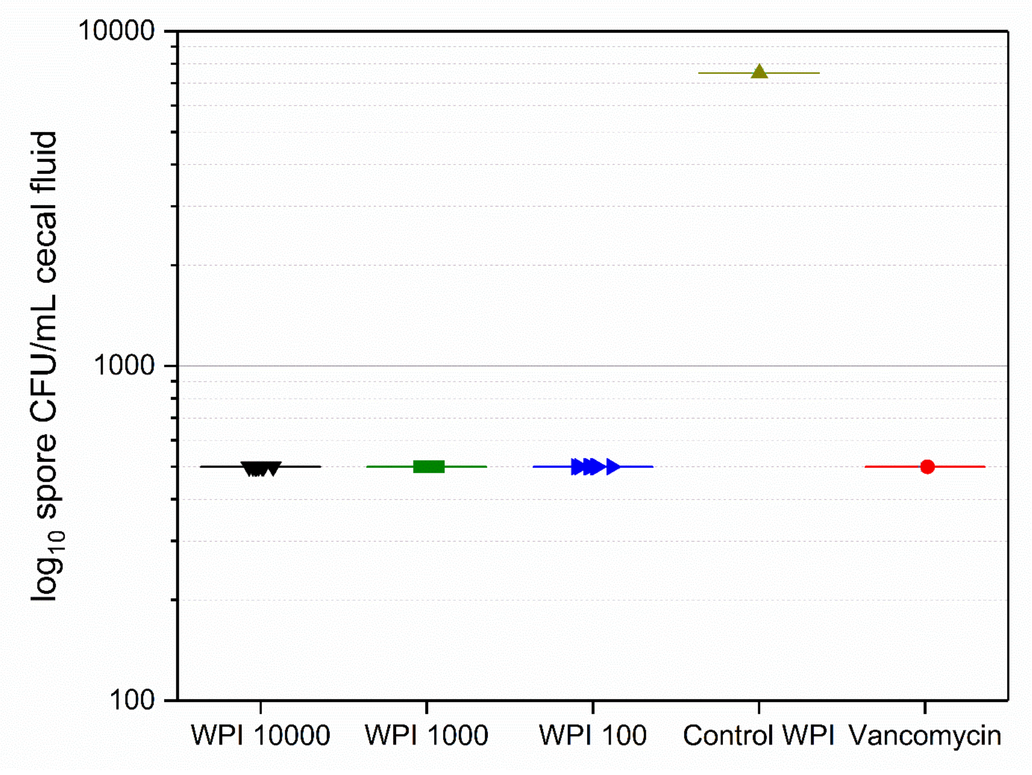

Figure S4 Surviving hamster were euthanized at the end of the study. The figure shows the spore counts CFU/ml in the cecal fluid at the day 21. Detection limit was 2.7 log_{10} CFU/ml.

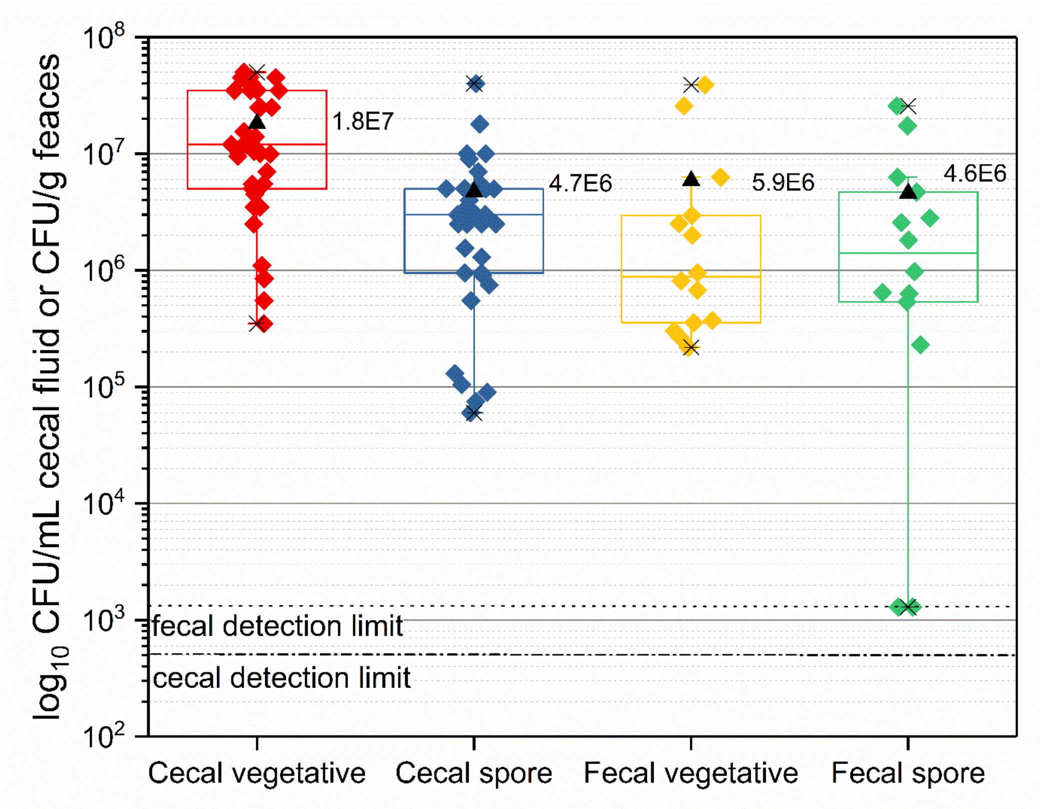

Figure S5 CFU/g feces or ml cecal fluid at the day of death of hamster who succumbed to the disease independent of applied treatment. The detection limit was 3.11 log10 CFU/g and 2.7 log_{10} CFU/ml for cecal samples, respectively.

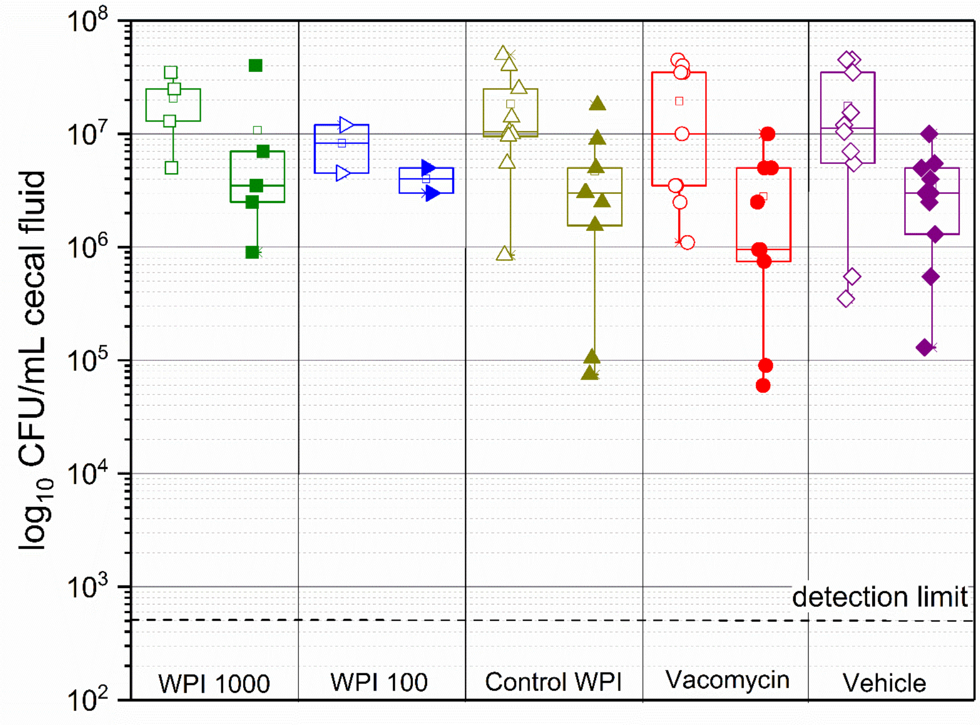

Figure S6 CFU/ml cecal fluid at the day of death of hamster who succumbed to the disease for the different applied treatments. Open symbols vegetative counts, filled symbols spore counts. The detection limit was 2.7 log_{10} CFU/ml

Table S1 Necropsy observation of cecum, ileum and colon

Group	USDA #	cecum obs.			ileum obs.			colon obs.		
		Overall appearance	Edema (present or absent)	Eechymotic hemorrhagic petechiae (present or absent) % coverage	Overall appearance	Edema (present or absent)	Eechymotic hemorrhagic petechiae (present or absent) % coverage	Overall appearance	Edema (present or absent)	Eechymotic hemorrhagic petechiae (present or absent) % coverage
**WPI 1000**	11622	Pinkish-tan color w/ hemorrhagic spots; swollen	present	present, >60%	Tan color w/ hemorrhagic spots; swollen	present	<10%	(ascending) Opaque white color; swollen	present	0
	11623	Yellow-tan color w/ hemorrhagic spots; swollen	present	present, <10%	Tan color w/ hemorrhagic spots; swollen	present	10%	(ascending) Opaque white color; swollen	present	0%
	11624	normal, brown color	none	none	normal, brown color	none	none	normal, brown color	none	none
	11625	normal, brown color	none	none	normal, brown color	none	none	normal, brown color	none	none
	11626	Yellow-red color w/ hemorrhagic spots; swollen	present	present, 40%	Tan color w/ hemorrhagic spots; swollen	present	<10%	(ascending) Opaque white color; swollen	present	0
	11627	Yellow-tan color w/ hemorrhagic spots; swollen	present	present, <10%	Tan color w/ hemorrhagic spots; swollen	present	10%	(ascending) Opaque white color; swollen	present	0%
	11628	normal, brown color	none	none	normal, brown color	none	none	normal, brown color	none	none
	11629	normal, brown color	none	none	normal, brown color	none	none	normal, brown color	none	none
	11630									
	11631	Yellow-tan color w/ hemorrhagic spots; swollen	present	present, <10%	Tan color w/ hemorrhagic spots; swollen	present	10%	(ascending) Opaque	present	0%

								white color; swollen		
WPI 100	11632	normal, brown color	none	none	normal, brown color	none	none	normal, brown color	none	none
	11633	normal, brown color	none	none	normal, brown color	none	none	normal, brown color	none	none
	11634	Pink-red color w/ hemorrhagic spots; swollen	present	present, 10%	Red-tan color w/ hemorrhagic spots; swollen	present	40%	(ascending) Opaque white color; swollen	present	0
	11635	normal, brown color	none	none	normal, brown color	none	none	normal, brown color	none	none
	11636	Yellow-tan color w/ hemorrhagic spots; swollen (contents are viscous, mucous-like)	present	present, <10%	Tan color w/ hemorrhagic spots; swollen	present	10%	(ascending) Opaque white color; swollen	present	0%
	11637	normal, brown color	none	none	normal, brown color	none	none	normal, brown color	none	none
	11638	normal, brown color	none	none	normal, brown color	none	none	normal, brown color	none	none
	11639	normal, brown color	none	none	normal, brown color	none	none	normal, brown color	none	none
	11640	normal, brown color	none	none	normal, brown color	none	none	normal, brown color	none	none
	11641	normal, brown color	none	none	normal, brown color	none	none	normal, brown color	none	none
control WPI	11642	normal, brown color	none	none	normal, brown color	none	none	normal, brown color	none	none
	11643	Pink-red color w/ hemorrhagic spots; swollen	present	present, 10%	Red-tan color w/ hemorrhagic spots; swollen	present	40%	(ascending) Opaque white color; swollen	present	0

	11644	Brown-red color w/ hemorrhagic spots; swollen	present	present, >70%	Tan color w/ hemorrhagic spots; swollen	present	<10%	(ascending) Opaque white color; swollen	present	0%
	11645	Brownish-tan color w/ hemorrhagic spots; swollen	present	present, >70%	Tan color w/ hemorrhagic spots; swollen	present	<10%	(ascending) Opaque white color; swollen	present	0%
	11646	Brownish-tan color w/ hemorrhagic spots; swollen	present	present, 40%	Tan color w/ hemorrhagic spots; swollen	present	<10%	(ascending) Opaque white color; swollen	present	0%
	11647	Yellow-tan color w/ hemorrhagic spots; swollen	present	present, 30%	Tan color w/ hemorrhagic spots; swollen	present	<10%	(ascending) Opaque white color; swollen	present	0%
	11648	Brown-red color w/ hemorrhagic spots; swollen	present	present, >70%	Tan color w/ hemorrhagic spots; swollen	present	<10%	(ascending) Opaque white color; swollen	present	0%
	11649	Yellow-tan color w/ hemorrhagic spots; swollen	present	present, <10%	Tan color w/ hemorrhagic spots; swollen	present	10%	(ascending) Opaque white color; swollen	present	0%
	11650	Dark brownish-red	present	present, 80%	Tan color w/ hemorrhagic spots; swollen	present	10%	(ascending) Opaque white color; swollen	present	0%
	11651	Brownish-tan color w/ hemorrhagic spots; swollen	present	present, >70%	Tan color w/ hemorrhagic spots; swollen	present	<10%	(ascending) Opaque white color; swollen	present	0
WPI 10000	11652	normal, brown color	none	none	normal, brown color	none	none	normal, brown color	none	none
	11653	normal, brown color	none	none	normal, brown color	none	none	normal, brown color	none	none

	11654	normal, brown color	none	none	normal, brown color	none	none	normal, brown color	none	none
	11655	normal, brown color	none	none	normal, brown color	none	none	normal, brown color	none	none
	11656	normal, brown color	none	none	normal, brown color	none	none	normal, brown color	none	none
	11657	normal, brown color	none	none	normal, brown color	none	none	normal, brown color	none	none
	11658	normal, brown color	none	none	normal, brown color	none	none	normal, brown color	none	none
	11659	normal, brown color	none	none	normal, brown color	none	none	normal, brown color	none	none
	11660	normal, brown color	none	none	normal, brown color	none	none	normal, brown color	none	none
	11661	normal, brown color	none	none	normal, brown color	none	none	normal, brown color	none	none
Vancomycin	11662	Brown-red color w/ hemorrhagic spots; swollen	present	present, >70%	Tan color w/ hemorrhagic spots; swollen	present	<10%	(ascending) Opaque white color; swollen	present	0%
	11663	Brown-red color w/ hemorrhagic spots; swollen	present	present, 50%	Tan color w/ hemorrhagic spots; swollen	present	<10%	(ascending) Opaque white color; swollen	present	0%
	11664	Brown-red color w/ hemorrhagic spots; swollen	present	present, >75%	Tan color w/ hemorrhagic spots; swollen	present	<10%	(ascending) Opaque white color; swollen	present	0%
	11665	Yellow-tan color w/ hemorrhagic spots; swollen	present	present, <10%	Tan color w/ hemorrhagic spots; swollen	present	10%	(ascending) Opaque white color; swollen	present	0%
	11666	normal, brown color	none	none	normal, brown color	none	none	normal, brown color	none	none

	11667	Light Brownish-tan color w/ hemorrhagic spots; swollen	present	present, 30%	Tan color w/ hemorrhagic spots; swollen	present	<10%	(ascending) Opaque white color; swollen	present	0%
	11668	Pink-red color w/ hemorrhagic spots; swollen	present	present, 10%	Red-tan color w/ hemorrhagic spots; swollen	present	40%	(ascending) Opaque white color; swollen	present	0
	11669	Brown-red color w/ hemorrhagic spots; swollen	present	present, >60%	Tan color w/ hemorrhagic spots; swollen	present	<10%	(ascending) Opaque white color; swollen	present	0%
	11670	Brown-red color w/ hemorrhagic spots; swollen	present	present, >80%	Tan color w/ hemorrhagic spots; swollen	present	<10%	(ascending) Opaque white color; swollen	present	0%
	11671	Pink-red color w/ hemorrhagic spots; swollen	present	present, 10%	Red-tan color w/ hemorrhagic spots; swollen	present	40%	(ascending) Opaque white color; swollen	present	0
Vehicle	11672	Yellow-tan color w/ hemorrhagic spots; swollen	present	present, <10%	Tan color w/ hemorrhagic spots; swollen	present	10%	(ascending) Opaque white color; swollen	present	0%
	11673	Yellow-tan color w/ hemorrhagic spots; swollen	present	present, <10%	Tan color w/ hemorrhagic spots; swollen	present	10%	(ascending) Opaque white color; swollen	present	0%
	11674	Brownish-tan color w/ hemorrhagic spots; swollen	present	present, 20-30%	Tan color w/ hemorrhagic spots; swollen	present	<10%	(ascending) Opaque white color; swollen	present	0%
	11675	Brown-red color w/ hemorrhagic spots; swollen	present	present, <10%	Tan color w/ hemorrhagic spots; swollen	present	<10%	(ascending) Opaque white color; swollen	present	0%

11676	Brownish-tan color w/ hemorrhagic spots; swollen	present	present, 20-30%	Tan color w/ hemorrhagic spots; swollen	present	<10%	(ascending) Opaque white color; swollen	present	0%
11677	Brown-red color w/ hemorrhagic spots; swollen	present	present, 50%	Tan color w/ hemorrhagic spots; swollen	present	<10%	(ascending) Opaque white color; swollen	present	0%
11678	Brownish-tan color w/ hemorrhagic spots; swollen	present	present, 30%	Tan color w/ hemorrhagic spots; swollen	present	10%	(ascending) Opaque white color; swollen	present	0%
11679	Light Brownish-tan color w/ hemorrhagic spots; swollen	present	present, 10-20%	Tan color w/ hemorrhagic spots; swollen	present	<10%	(ascending) Opaque white color; swollen	present	0%
11680	Yellow-tan color w/ hemorrhagic spots; swollen	present	present, <10%	Tan color w/ hemorrhagic spots; swollen	present	10%	(ascending) Opaque white color; swollen	present	0%
11681	Brownish-tan color w/ hemorrhagic spots; swollen	present	present, <60%	Tan color w/ hemorrhagic spots; swollen	present	10%	(ascending) Opaque white color; swollen	present	0%
